# Effects of Yupingfeng Polysaccharides as Feed Supplement on Immune Function and Intestinal Microbiome in Chickens

**DOI:** 10.3390/microorganisms11112774

**Published:** 2023-11-15

**Authors:** Wendan Zheng, Yuling Guan, Bo Wu

**Affiliations:** Guangdong Provincial Key Laboratory of Animal Molecular Design and Precise Breeding, School of Life Science and Engineering, Foshan University, Foshan 528000, China

**Keywords:** Yupingfeng polysaccharide, microflora, growth performance, immune, antioxidant balance

## Abstract

The health of chicks is closely related to their productivity. Yupingfeng polysaccharide (YPF-P) is a kind of water-soluble polysaccharide extracted from Yupingfeng powder; it has high pharmacological activity and can be used as a potential substitute for antibiotics to improve the health of chicks. This study aimed to investigate the effects of YPF-P on immune performance, the duodenum, and the cecal microflora of chicks. All chickens (4224) were randomly distributed into four groups (eight replicas/group, 132 hens/replica). The control group was fed a basal diet (0 g/kg YPF-P), while the experimental groups were fed basal diets supplemented with 1, 2, or 4 g/kg YPF-P. The results showed that YPF-P significantly increased the thymus index (*p* < 0.05). The content of total antioxidant capacity (T-AOC), superoxide dismutase (SOD), glutathione peroxidase (GSH-Px), immunoglobulin A (IgA), and IgG and immunoglobulin M (IgM) was upregulated in the serum by YPF-P (*p* < 0.05). YPF-P decreased the content of malondialdehyde (MDA) (*p* < 0.05). Further, 16S rRNA sequencing showed that 2 g/kg YPF-P modulated the predominant duodenum and cecal microbial community structure, which increased the number of *Faecalibacterium*, *Megamonas*, *Bacteroides*, *Alistipes*, *NK4A214_group*, and *Enterococcus*. In conclusion, YPF-P ameliorated the growth performance of chicks by regulating serum immune and antioxidant balance, as well as the intestinal microbiota.

## 1. Introduction

The chicken intestine is the largest digestive organ and the largest immune organ in chickens. Intestinal health is very important in the growth and development of chickens. Especially in the chick stage, the early colonization of intestinal microorganisms will be changed due to various factors, which will further affect the production performance and disease resistance of chicks [1,2]. In addition, the intestinal flora of chickens is also vulnerable to stress interference caused by various external factors, and oxidative stress can lead to intestinal inflammation and other diseases [3]. It is the unremitting pursuit of animal husbandry to ensure healthy intestinal microflora during the growth and development of livestock and poultry. Therefore, the development of new, efficient, green, and safe feed supplements plays a vital role in improving poultry’s immunity and intestinal flora.

The rapid development of the global meat and poultry industry cannot be separated from the role of antibiotics. The addition of antibiotics in meat poultry production has reduced the occurrence of poultry diseases to a certain extent and enhanced poultry’s immunity, and has improved the feed utilization rate, reduced the mortality rate, and significantly improved the health of poultry. People are gradually realizing that antibiotics can kill pathogenic bacteria and affect poultry’s immunity, thus increasing the economic benefits of breeding [4]. As a result, people have been using antibiotics continuously in poultry production. The problem of drug resistance caused by the abuse of antibiotics has had a massive impact on human life and health and food safety, which has attracted everyone’s attention [5]. The Ministry of Agriculture and Rural Affairs issued Announcement No. 194, announcing that from 1 July 2020, farmers are forbidden to add antibiotics to feed, to reduce the harm of antibiotic abuse and maintain the safety of animal-derived food [6]. A total ban on antibiotics means that animals need other antibiotic substitutes to protect their health. Animal husbandry urgently needs the emergence of antibiotic substitutes to enhance animal disease resistance in livestock and poultry production.

In recent years, plant extracts have entered the feed additive market as a green and healthy additive. Because of their efficient pharmacological activity, they can enhance the immunity, disease resistance, and oxidation resistance of animals to a certain extent; regulate the intestinal flora of animals, thus promoting the growth and development of animals and ultimately improving the economic benefits of breeding; and are often used in intensive poultry breeding [1,7,8]. Yupingfeng powder is a kind of traditional Chinese medicine prescription [9] that is prepared using three Chinese herbal medicines, namely, *Astragalus membranaceus* (Fisch.) Bunge (AR, Huangqi), *Atractylodes macrocephala* Koidz (AMK, Baizhu), and *Saposhnikovia divaricata* (SD, Fangfeng), in a certain proportion. It has the functions of invigorating qi, consolidating the exterior, and arresting sweating in traditional Chinese medicine treatment [10]. Its active component, Yupingfeng polysaccharide (YPF-P), has certain effects on immune regulation in hepatocellular carcinoma [11], and can improve antioxidant capacity in vivo on the basis of scavenging free radicals, thus reducing stress damage [12] and promoting growth and development [13]. Some researchers have found that adding 0.12% YPF-P to the basic feed of grass carp under heat stress could improve the activity of antioxidant enzymes in fish to some extent and alleviate the liver damage caused by heat stress [14]. YPF-P can also resist cancer by inhibiting bone marrow-derived cell proliferation in mice, inhibiting cell proliferation, and promoting cell apoptosis to reshape the mouse tumor microenvironment [15]. The polysaccharide isolated from YPF (YPF-PS) can promote the proliferation of poultry lymphocytes and increase the antibody titer that defines HI (hemagglutination inhibition) in poultry serum, indicating that YPF has a robust immune effect [16]. The above studies all indicate that YPF-P plays a certain role in oxidative stress, cancer, and immunity in animals, thus enhancing the immunity of animals. It shows that YPF-P benefits animals and is a kind of green plant additive worth using.

Adding plant polysaccharides to chicken feed can improve the intestinal health of broilers, promote the growth and development of intestinal villi and intestinal metabolism, and replace antibiotics to enhance the immune regulation function of the body [17]. But there are few reports about YPF-P replacing antibiotics in poultry and other clinical research [18]. In this study, a 1 g/kg, 2 g/kg, and 4 g/kg amount of YPF-P was added to chick feed to explore the effects of YPF-P on the serum immunity and intestinal morphology of chicks. 16S rRNA sequencing of the duodenum content and cecum content was carried out to detect the structural composition and abundance changes of the intestinal flora. This study provides a reference for efficiently utilizing YPF-P as a green and safe feed additive in poultry production.

## 2. Materials and Methods

### 2.1. Materials

Yupingfeng polysaccharide (YPF-P) was obtained from the Dezhong National Medicine Group (Foshan) Drug Co., Ltd., Guangdong, China. Preparation Yupingfeng polysaccharides was carried out according to reference [19]. In the preparation of Yupingfeng polysaccharide, the amounts of crude drugs (in powder) of AR, AKM, and SD were weighed according to the weight ratio of 3:1:1, respectively. The ratio of material to liquid was 1:16, the extraction temperature was 90 °C, the extraction time was 90 min, the extractions were carried out 3 times, and the gauze was filtered. After the precipitation was complete, the supernatant was taken for suction filtration, the filtrate was concentrated using a rotary evaporator (RE-201D, Yuhua instrument manufacturing Co., Ltd., Zhengzhou, China), absolute ethanol was added for 12 h, and the precipitate was purified using macroporous resin. The concentrated solution obtained by boiling twice was filtered, and the refined Yupingfeng polysaccharide was made into powder at low temperature via vacuum lyophilization (Labconco, Kansas City, MO, USA) and stored at 4 °C. The polysaccharide content of Yupingfeng determined via the phenol sulfuric acid method was 90.11%. According to HPLC (high-performance liquid chromatography) analysis, its monosaccharide composition mainly included D-mannose, D-ribose, rhamnose, D-galacturonic acid, D-glucose, D-galactose, arabinose, etc. The molar ratio of monosaccharides in these 7 substances was 4.64:1:5.88:2.40:120:42.9:69.5. The contents of total reducing sugar, crude protein, crude fat, crude ash, moisture, and crude fiber were 34.20%, 18.52%, 0.71%, 8.86%, 8.21%, and 0.67%, respectively.

### 2.2. Animal Treatments and Design

A total of 4224 three-day-old Qingyuan partridge chickens were acquired from Guangdong Tiannong Food Group Co., Ltd., Qingyuan poultry farm (Qingyuan, China). All chickens were individually weighed and stochastically allocated into four treatment groups (8 replicates, each with 132 chickens). The chickens in the control group were fed the basal diet (0 g/kg YPF-P). The treatment groups were as follows: a basal diet supplemented with 1 g/kg YPF-P (1 g/kg YPF-P), a basal diet supplemented with 2 g/kg YPF-P (2 g/kg YPF-P), and a basal diet supplemented with 4 g/kg YPF-P (4 g/kg YPF-P). Throughout the 34-day experiment, all chickens were placed in three-layer battery cages (100 cm length × 60 cm width × 35 cm height) and could freely access food and water. In the first week, the room temperature was 35 °C and was decreased by 2 °C each week until the room temperature was maintained at about 25 °C. During the experiment, all chicks were vaccinated against Newcastle disease and avian influenza at one day old, seven days old, and twenty-one days old in time according to chicken breeding standards [20]. According to the NRC (National Research Council, 1998) recommendations, the basic diet can meet the nutritional needs of chicks during the starter (days 1 to 21) and grower (days 22 to 42) periods (Table 1). This study was performed strictly per the Animal Management Rules of the Ministry of Health of the People’s Republic of China and approved by the Animal Care and Use Committee of Foshan University (Foshan, China). Approval Code: FOSU 2022-307. After the experiment, chickens were euthanatized.

### 2.3. Immune Organ Indexes

All chickens in each pen were weighed every week. After the experiment, 8 chickens with similar weights and health statuses were randomly selected from each group (we chose a chicken from each of the 8 replicates in each group). Each chicken was bled via its neck until death, and we obtained the spleen, thymus, and bursa of Fabricius (BF), and then, recorded its weight. The index of spleen, thymus, and BF was calculated according to the following formula:

Index (g/kg) = immune organs weight/body weight

### 2.4. Serum Biochemical Analysis

At day 34 of the experiment, 8 chickens with similar weights and health were randomly selected from each group, and 5 mL blood was collected from the pterygoid vein and negotiated to glass tubes without anticoagulants; then it was centrifuged at 3500 r/min for 15 min. Serum was obtained and stored at −20 °C for biochemical analysis.

The serum contents of immunoglobulin A (IgA), IgG, and immunoglobulin M (IgM) were detected using specific ELISA kits purchased from Shanghai Yuanju Biotechnology Center (Shanghai, China). Total antioxidant capacity (T-AOC), superoxide dismutase (SOD), malondialdehyde (MDA), and glutathione peroxidase (GSH-Px) in the serum were measured using relevant reagent kits from the Nanjing Jiancheng Bioengineering Institute (Nanjing, China).

### 2.5. Duodenum and Cecum Morphology Analysis

The intestinal morphology was analyzed by measuring the villus height (VH) and crypt depth (CD) of the duodenum and cecum. According to the experimental results of serum biochemical analysis, it is known that the dosage of YPF-P of 2 g/kg was the best in this experiment, so we chose the 2 g/kg YPF-P group and 0 g/kg group to compare the intestinal morphology. The cecum and duodenum segments (1 cm, *n* = 8) of chickens were collected after the experiment. Duodenum and cecum segments were fixed in 4% paraformaldehyde solution at room temperature until analysis. Samples of duodenum and cecum fixed in 4% paraformaldehyde solution were dehydrated with alcohol and embedded in paraffin. Using a paraffin slicing machine (MCT-001, HistoCore BIOCUT, Wetzlar, Deutschland), the embedded paraffin blocks were cut into 4 mm thicknesses, fixed on a glass slide, and stained with hematoxylin–eosin (H-E). The VH and CD were observed under an optical microscope, and then, the ratio VCR of VH to CD was calculated. The intestinal cross-section of each chicken was measured at 3 points randomly, and the average value was taken.

### 2.6. S rRNA Sequencing and Analysis

According to the results of serum biochemical analysis, we selected the 2 g/kg YPF-P group with the best performance in the YPF-P group to compare with the 0 g/kg group, so as to detect the influence of YPF-P on the intestinal flora of chicks. After the experiment, 8 chickens in each group were randomly selected so that we could take the contents of the duodenum and cecum for 16S rRNA sequencing and analysis. 

Total genomic DNA was extracted from duodenum and cecum samples using the T Guide S96 Magnetic Soil/Stool DNA Kit (Tiangen Biotech (Beijing) Co., Ltd., Beijing, China) according to manufacturer’s instructions. The quality and quantity of the extracted DNA were examined using electrophoresis on 1.8% agarose gel, and the DNA concentration and purity were determined using a Nano Drop 2000 UV-Vis spectrophotometer (Thermo Scientific, Wilmington, DE, USA). The hypervariable regions V3-V4 of the bacterial 16S rRNA gene were amplified with primer pairs 338F (5′-ACTCCTACGGGAGGCAGCA-3′) and 806R (5′-GGACTACHVGGGTWTCTAAT-3′). Both the forward and reverse 16S primers were tailed with sample-specific Illumina index sequences to allow for deep sequencing [21]. The PCR was performed in a total reaction volume of 10 μL containing the following: DNA template 5–50 ng, forward primer (10 μM) 0.3 μL, reverse primer (10 μM) 0.3 μL, KOD FX Neo Buffer 5 μL, dNTP (2 mM each) 2 μL, KOD FX Neo 0.2 μL, and finally, ddH2O up to 20 μL. After initial denaturation at 95 °C for 5 min, followed by 20 cycles of denaturation at 95 °C for 30 s, annealing at 50 °C for 30 s, and extension at 72 °C for 40 s, and a final step at 72 °C for 7 min. The amplified products were purified using an Omega DNA purification kit (Omega Inc., Norcross, GA, USA) and quantified using Qsep-400 (BiOptic, Inc., New Taipei City, Taiwan, ROC). The amplicon library was paired-end sequenced (2 × 250) using an Illumina novaseq6000 (Beijing Biomarker Technologies Co., Ltd., Beijing, China). 

### 2.7. Statistical Analysis

The test data for immune organ indexes, immune factors, antioxidant indexes, and morphological analyses are presented as “mean ± SEM” and were analyzed using SPSS (SPSS 20.0, SPSS, Chicago, IL, USA). All the data conform to a normal distribution. So, the data were analyzed via one-way ANOVA, and Tukey’s tests were performed to evaluate the between-group differences. When *p* < 0.05, the result is considered statistically significant. We used GraphPad Prism 9.0 (GraphPad Software Inc., San Diego, CA, USA) to produce the figures. The data analysis and figure preparation for the microbiome were performed using BMKCloud (www.biocloud.net accessed on 16 February 2023).

Correlation assays were conducted between the gut microbes (at the genus level), immune factors, and antioxidant indexes. Data were input into https://www.omicstudio.cn (accessed on 27 September 2023) to calculate the correlation coefficients based on Spearman’s correlation distance. Heatmaps were prepared using OmicStudio to assess the bivariate relationships between variables. * *p* < 0.05, ** *p* < 0.01.

## 3. Results

### 3.1. Influence of Yupingfeng Polysaccharides on the Index of Immune Organs

**Figure 1:**
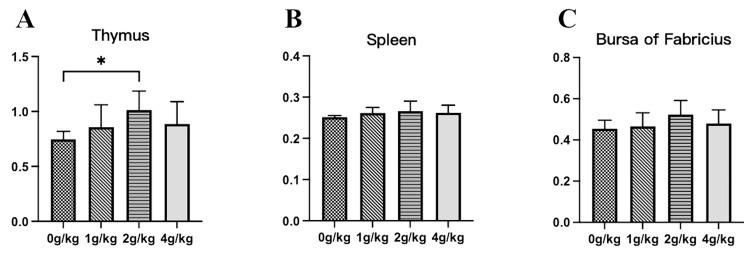
Effects of YPF-P treatment on immune organs: (**A**) thymus, (**B**) spleen, (**C**) bursa of Fabricius. The data are presented as mean ± SEM. The bars with * in the figure indicate significant differences (*p* < 0.05).

The result of the immune organ index is shown in Figure 1. During the YPF-P period, the results for the spleen and bursa of Fabricius exhibited no statistical significance among the four groups. However, compared to the control group (0 g/kg), the results showed that the thymus index of the 2 g/kg YPF-P group was significantly increased (*p* = 0.044) (Figure 1A).

### 3.2. Influence of Yupingfeng Polysaccharides on Immune Level and Serum Antioxidant Indexes

The effects of YPF-P on the serum antioxidant parameters in early-stage chicks are shown in Table 2. Compared with the control group (0 g/kg), the 2 g/kg YPF-P-group chicks exhibited significantly higher content of serum T-AOC (*p* < 0.05). The dietary supplementation with YPF-P significantly increased the serum concentration of SOD (*p* < 0.05). The level of serum GSH-PX in both the 2 g/kg YPF-P-group and the 4 g/kg YPF-P-group chicks was significantly higher than that in the control group (0 g/kg) (*p* < 0.05). Moreover, the supplementation with YPF-P significantly decreased the content of serum the MDA in chicks (*p* < 0.05).

**Figure 2:**
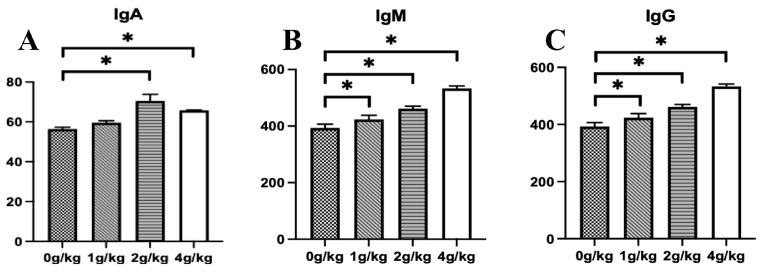
Effects of YPF-P treatment on immune level: (**A**) IgA, (**B**) IgG, (**C**) IgM. The data are presented as mean ± SEM. The bars with * in the figure indicate significant differences (*p* < 0.05).

The effects of YPF-P on the immune level parameters in early-stage chicks are shown in Figure 2. Compared with the control group (0 g/kg), the levels of the immune factors IgA, IgM, and IgG in the serum of chicks were significantly increased in both the 2 g/kg YPF-P group and the 4 g/kg YPF-P group (*p* < 0.05) (Figure 2A–C). The level of Ig A in the 1 g/kg YPF-P group was not significantly higher than that in the control group (Figure 2A), but the levels of IgM and IgG were significantly higher than that in the control group (0 g/kg) (Figure 2B,C).

### 3.3. Influence of Yupingfeng Polysaccharides on Duodenum and Cecum Intestinal Morphology

**Figure 3:**
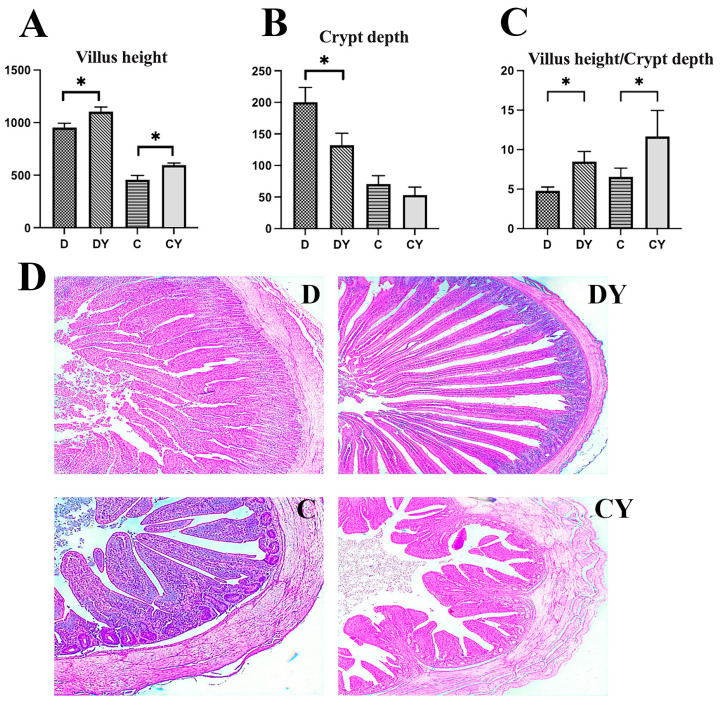
Effects of YPF-P treatment on duodenum and cecum intestinal morphology: (**A**) Villus height. (**B**) Crypt depth. (**C**) Villus height/crypt depth. (**D**) Representative images of hematoxylin–eosin (H-E)-stained duodenum and cecum tissue. The bars with * in the (**A**–**C**) indicate significant differences (*p* < 0.05). (**D**)—(C is the cecal control group (0 g/kg), and CY is the cecal 2 g/kg YPF-P addition group. D is the duodenal control group (0 g/kg), and DY is the duodenal 2 g/kg YPF-P addition group).

The duodenum and cecum morphologies of broiler chicks are presented in Figure 3. Compared with controls, the addition of YPF-P significantly increased (*p* < 0.05) the villus height (VH) and villus height/crypt depth ratio (V/C) (Figure 3A,C). The YPF-P supplementation significantly reduced the chicks’ crypt depth (VCR) (Figure 3B). The HE-stained duodenum and cecum sections showed that the intestinal morphology was normal, the intestinal villi were arranged neatly, and there were no lesions in the four groups (Figure 3D). 

### 3.4. Influence of Yupingfeng Polysaccharides on Duodenum and Cecal Microflora

**Figure 4:**
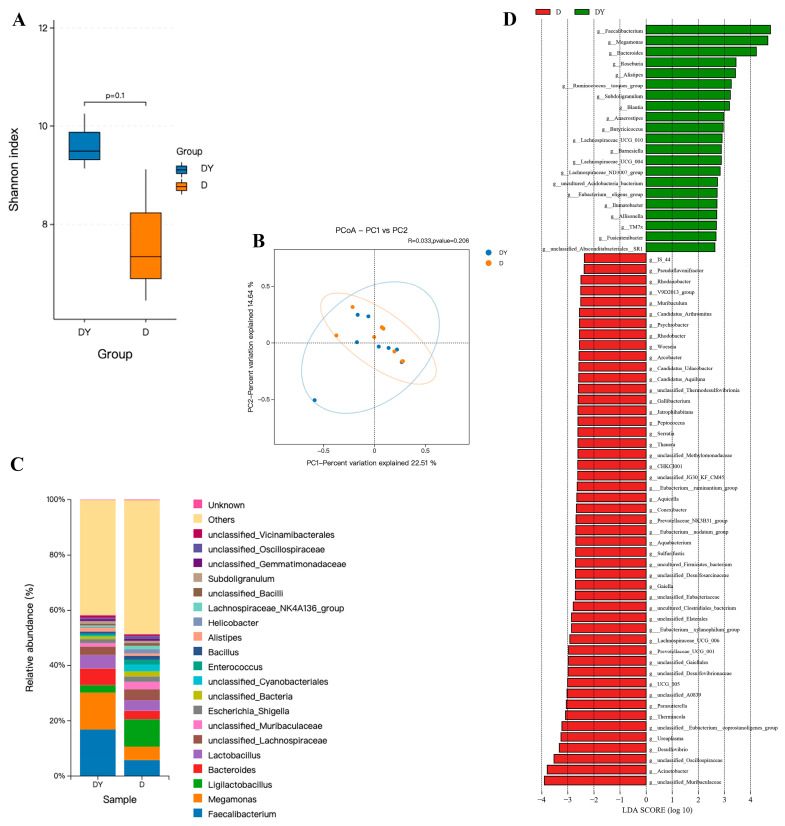
Impacts of YPF-P on duodenal microbiota of chicks: (**A**) Alpha diversity of duodenal microbiota in different groups. Data were analyzed using nonparametric Wilcoxon’s test. (**B**) Principal coordinate analysis of duodenal microbiota in different groups. According to the Bray–Curtis distance, the PCoA diagram was generated using abundance data, and the statistical significance was measured via adonis analysis. (**C**) Alteration of duodenal microbiota on the genus level. (**D**) LEfSe analysis of duodenal microbiota (LDA score is greater than 2).

**Figure 5:**
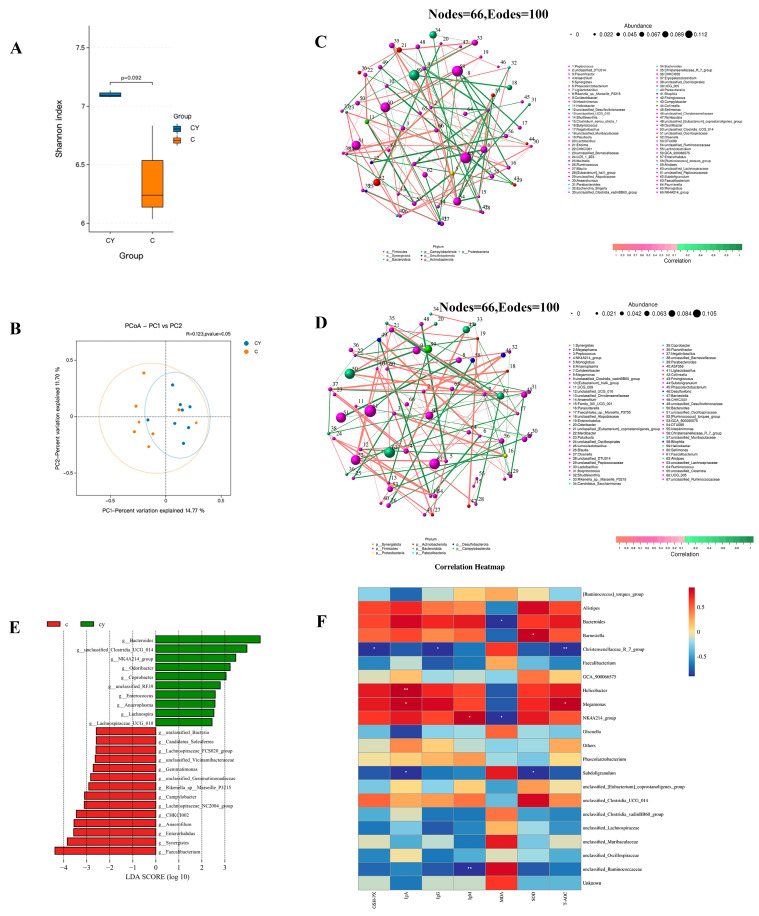
Impacts of YPF-P on cecal microbiota of chicks: (**A**) Alpha diversity of cecal microbiota in different groups. Data were analyzed using a nonparametric Kruskal–Wallis test. (**B**) PCoA of cecal microbiota in different groups. According to the Bray–Curtis distance, the PCoA diagram was generated using OTU abundance data, and the statistical significance was measured via adonis analysis. (**C**) Analysis of symbiotic network of intestinal microorganisms based on core genus (average relative abundance in the C groups > 0.1%). Nodes of different colors represent different phylum levels. Different colors with edges represent significant correlation, red represents positive correlation, and green represents negative correlation (*p* < 0.05). (**D**) Analysis of symbiotic network of intestinal microorganisms based on core genus (average relative abundance in the CY groups > 0.1%). (**E**) LEfSe analysis of cecal microbiota (LDA score is greater than 2). (**F**) Spearman’s correlation analysis between immune and antioxidant indexes and cecal microbes at the genus level: *p* < 0.05. Red represents positive correlations and blue represents negative correlations. * *p* < 0.05, ** *p* < 0.01. *n* = 8.

In order to evaluate the effect of YPF-P on the intestinal flora of chicks, the contents of the cecum and duodenum of laying hens were sequenced using 16S rRNA. The results showed that YPF-P (DY) had no significant effect on the α-diversity (as analyzed using the Shannon index) and β-diversity (as determined via PCoA analysis) of the duodenal microflora (Figure 4A,B). But at the genus level, compared with the control group (C), YPF-P (DY) increased several beneficial bacteria, such as *Faecalibacterium*, *Megamonas*, *Bacteroides*, *Alistipes*, and Lactobacillus (Figure 4C). The subsequent linear discriminant analysis (LDA) effect size (LEfSe) results showed that there were obvious differences in duodenal bacterial communities among the different groups (Figure 4D). The dominant strains in the duodenal control group (D) were *unclassified_Muribaculaceae*, *Acinetobacter*, *unclassified_Oscillospiraceae*, and *Desulfovibrio*, and the dominant strains in YPF-P addition group (DY) were *Faecalibacterium*, *Alistipes*, *Roseburia*, *Megamonas*, and *Bacteroides*. In contrast, Shannon analysis also showed that there was no significant difference in cecum flora between the control group (C) and YPF-P group (CY), but principal coordinate analysis (PCoA) showed that there was a significant difference in cecum flora between the two groups (Figure 5A,B). The co-occurrence network analysis based on the relative abundance of genera showed that there were 66 nodes and 100 edges in the control group (C), and the YPF-P group (CY) had 67 nodes and 100 edges (Figure 5C,D). The core genera of the control group (C) and the YPF-P group (CY) were contained in seven phyla and eight phyla, respectively, which shows that the bacterial network of YPF-P is not more complicated than that of the control group (C). The linear discriminant analysis (LDA) effect size (LEfSe) results showed that there were obvious differences in cecal bacterial communities among different groups (Figure 5E). At the genus level, compared with the control group (C), YPF-P (CY) increased the number of beneficial bacteria such as *Bacteroides*, *NK4A214_group* and *Enterococcus*, and decreased the number of *Enterarhabdus*, *Synergistes*, *Campylobacter*, and *Faecalibacterium* (Figure 5E). Further correlation analysis showed that *Megamonas* was positively correlated with IgA and T-AOC, *K4A214_group* was positively correlated with IgM, and *NK4A214_group* and *Bacteroides* were negatively correlated with MDA (Figure 5F). To sum up, these data indicate that YPF-P may enhance the immune performance of chicks by regulating the cecal and duodenal microflora.

## 4. Discussion

Immune function is a crucial defense barrier for maintaining healthy poultry production and important to promote the efficient production of the modern poultry industry. Poultry immune organs can be divided into central and peripheral immune organs according to their functions. The thymus, which plays a role in the differentiation and maturation of T cells, is one of the most essential immune organs in poultry and can participate in humoral immunity and cellular immunity. The spleen, as a peripheral immune organ, can produce many lymphocytes. The bursa of Fabricius is mainly involved in humoral immunity, and it is the primary place for the development and proliferation of immunocompetent B cells [22]. The organ indexes of these three organs are closely related to the immunity of chicks [23]. The immune level of chickens is affected by the development of immune organs. The increase in the relative weight of immune organs means that the immune level of the body also increases [24]. This study showed that adding a 2 g/kg amount of YPF-P to the feed of chicks can increase the thymus index of chicks. Immune organs and serum immune factors are critical indicators to test the immune function of poultry, and the levels of IgA, IgG, and IgM in serum are the leading indicators that reflect the humoral immune level of animals. Therefore, we further studied the effects of immune factors in serum on the early immune function of chicks. This study found that compared with the control group (0 g/kg), the YPF-P group had significantly increased serum levels of IgA, IgG, and IgM, and the effects of adding 2 g/kg and 4 g/kg YPF-P were particularly obvious. Some researchers found that Yupingfeng polysaccharide decoction can significantly improve the serum IgG and IgM levels in immunosuppressed mice and improve the immune function of mice [25]. Other studies have shown that adjuvant therapy with Yupingfeng granules can improve the overall clinical remission rate of recurrent respiratory tract infections (RRTIs) in children and increase the levels of the serum immunoglobulins IgA, IgM, and IgG, thus reducing the recurrence rate of infection and improving children’s immunity [26]. This is consistent with our research results. The above results indicate that YPF-P can replace antibiotics to a certain extent and enhance the immune function of animals. The 2 g/kg effect in this experiment has the optimum effect. 

Oxidative stress is an important factor that can destroy mucosal barrier function [27]. Oxidative stress in poultry will lead to the accumulation of anaerobic free radicals (ROS) in the body, and the peroxidation of nutrients in the body will increase energy waste and destroy redox balance [28]. Antioxidant substances derived from plants can neutralize anaerobic free radicals (ROS), significantly reduce peroxidation damage, and reduce the peroxidation effect on nutrients [29]. Malondialdehyde (MDA), the final product of lipid peroxidation in serum, is a biomarker used to measure the level of oxidative stress. At the same time, total antioxidant capacity (T-AOC) and glutathione peroxidase (GSH-PX) activity are usually used as the primary markers to detect serum antioxidant capacity [14]. Studies have shown that supplementation with Lonicera hypoglauca and Scutellaria baicalensis extract in the diet can improve oxidative stress by increasing SOD level and reducing MDA level in the serum of colitis mice [30]. Other studies have shown that polysaccharides extracted from salvia miltiorrhiza residue can increase the levels of T-AOC and SOD in the serum of weaned piglets, and reduce the level of MDA in the serum [31]. Feeding with 100 mg/kg *Antrodia cinnamomea* polysaccharide can increase the activities of T-AOC, GSH-Px, and SOD, and reduce the content of MDA, in slow-growing broilers stimulated by Lipopolysaccharides [32]. Polysaccharides extracted from Morchella sextelata also have strong antioxidant activity, and can be used as antioxidants to help the antioxidant defense system repair oxidative damage [33]. This study found that YPF-P significantly increased the contents of T-AOC, SOD, and GSH-PX in the serum and significantly decreased the content of MDA to enhance the antioxidant capacity of poultry. Studies have found a significant correlation between inflammation and oxidative stress in poultry, and more severe oxidative stress suffered by poultry will aggravate the inflammatory response in poultry [34]. Our results showed that YPF-P has a good antioxidant effect, which can reduce the risk of inflammation in poultry and reduce excessive waste in the process of energy use in poultry [35]. 

In modern poultry production, intestinal health is related to production performance. The intestine maintains the homeostasis of the body’s internal environment and is an immune barrier that protects the body. The morphological structure of poultry’s small intestine plays a vital role in the digestion of nutrients and is the principal place where most nutrients are consumed [36]. Intact intestinal morphology is a critical factor in preventing the invasion of pathogenic microorganisms [37]. Many studies show that plant polysaccharides can enhance intestinal function and improve intestinal immunity by improving intestinal morphology, thus maintaining the healthy growth of poultry and ultimately improving economic benefits [38]. Many researchers have used the V/C ratio of intestinal VH to crypt VCR to estimate the rate of nutrient absorption by the small intestine [39]. The larger the value, the stronger the animal’s ability to absorb nutrients, which is one of the essential indexes reflecting the digestive and absorption function of the body [40]. It was found that adding different concentrations of Yupingfeng polysaccharide compound probiotics to the primary feed of snakeheads can improve the V/C value of Channaargus [41]. In this study, the addition of 2 g/kg YPF-P could improve the mucosal morphology of the small intestine and significantly increased the villus height (VH) and villus height/crypt depth ratio (V/C) (*p* < 0.05), so we speculated that YPF-P could enhance intestinal health by improving the intestinal morphology of animals, thereby improving the production performance of poultry. Normal gastrointestinal microflora is very important for the growth and development of organisms, affecting the morphological development of intestinal villi and crypts and the absorption of nutrients [42], so we further analyzed the intestinal microflora.

The gastrointestinal health of poultry is not only related to the intestinal morphology, but also to the intestine’s microbial community. Studies have shown the intestinal flora is closely related to substance metabolism, growth, and development, and participates in energy metabolism, digestion and absorption, maintaining energy balance, and regulating immunity [43]. Intestinal microorganisms are also related to intestinal structure, intestinal immune barrier function, and intestinal inflammation [44]. This study found that in the cecum and duodenum, the flora abundance of the YPF-P group was higher than that of the control group. Previous studies have shown that adding Yupingfeng polysaccharide synbiotics to rex rabbits’ primary diet can significantly improve the intestinal flora abundance, structure, and intestinal immune barrier function of rex rabbits [45]. Moreover, studies have shown that the abundance of intestinal flora can enhance intestinal immune function [46] and reduce the risk of intestinal infection to a certain extent [47]. This is consistent with the results of our study. We think that YPF-P inhibits the production and proliferation of harmful bacteria in the duodenum and cecum. In this study, we found that the addition of YPF-P increased the number of *Bacteroidota* in the cecum and duodenum at the genus level. Studies have found that *Bacteroidota* can improve the intestinal mucosal barrier by reducing intestinal inflammation and can convert carbohydrates into butyrate [48]. LEfSe analysis shows that addition of YPF-P increased the number of dominant bacteria such as *Bacteroides*, *Lactobacillus*, *Megamonas*, and *Barnesiella* in the duodenum and the number of *NK4A214_group* and *Enterococcus* in the cecum at the genus level. LEfSe analysis also showed that the dominant flora of YPF-P is quite different from that of the control group (0 g/kg). The dominant bacteria in the YPF-P group are mainly intestinal beneficial bacteria, while the dominant bacteria in the control group are mainly pathogenic. For example, *Campylobacter*, a dominant bacteria in the cecum control group, is a pathogenic bacteria. Poultry is a *Campylobacter* species in a natural reservoir, and broilers are often colonized. It is estimated that 80% of human *Campylobacter* cases are caused by poultry hosts, and people infected with *Campylobacter* will develop gastroenteritis [49]. The addition of YPF-P reduced the flora abundance of *Campylobacter* in the cecum at the genus level. *Bacteroides*, the dominant bacteria in the YPF-P group of the cecum at the genus level, can provide nutrition for other microorganisms and protect the body from intestinal pathogens [50]. In addition, *Barnesiella*, *Faecalibacterium*, and *Bacteroides* were found to be related to the level of SCFAs in the intestine, and they could combine *Odoribacter* and *Parabacteroides* to produce butyric acid with immunomodulatory and anti-inflammatory effects by regulating the homeostasis of intestinal T cells [51]. LESse analysis of the duodenum at genus level also showed that the abundance of these three bacteria increased after YPF-P was added. Therefore, we believe that YPF-P can improve intestinal immune function, mainly by increasing the content of beneficial bacteria and reducing the content of harmful bacteria, to maintain intestinal health, reduce intestinal inflammation, play an immunomodulatory role, and improve growth performance.

## 5. Conclusions

In summary, adding YPF-P to chick feed can replace antibiotics to some extent and improve the thymus index of immune organs by increasing the levels of IgA, IgG, and IgM in broiler serum, thus enhancing immunity. By increasing the contents of T-AOC, SOD, and GSH-PX in the serum, the content of MDA was decreased, and the antioxidant capacity was increased. Additionally, it can improve the intestinal morphology, optimize the distribution of the intestinal flora, and provide a good redox environment and endogenous intestinal homeostasis for microbial communities, and can ensure that chicks are in a healthy growth state. Moreover, the content of Yupingfeng polysaccharide in feed is low (only 0.002%), and the cost is almost negligible. The lower dosage can also improve the immunity and antioxidant capacity of chicks and improve the distribution of the intestinal flora. The above results show that YPF-P can be used as a potential tool to keep chickens healthy, improve production performance, and increase economic benefits.

## Figures and Tables

**Table 1:** Composition and nutrient levels of the basal diet.

Ingredients (%)	d 1 to 21	d 22 to 42
Corn	67.2	71.4
Soybean meal	26.0	21.8
Fish meal	2.0	2.0
Meat meal	1.0	1.0
CaHPO_4_	1.5	1.5
Limestone	1.0	1.0
NaCl	0.3	0.3
Premix ^1^	1.0	1.0
Total	100.00	100.00
Chemical Composition Calculated (%)		
ME/(MJ/kg) ^2^	13.51	13.53
CP	19.71	18.11
P	0.80	0.79
Ca	1.10	1.09
Met	0.31	0.29
Lys	1.02	0.91

^1^ Provided per kilogram of diet: VA1 2000 IU, VB 13.5 mg, VB2 8 mg, VD3 3000 IU, VB12 25 mg, VE20 IU, riboflavin 7.5 mg, pantothenic acid 18.5 mg, biotin 0.25 mg, Fe 100 mg, Zn 40 mg, Cu 8 mg, Mn 30 mg, I 0.3 mg. ^2^ ME was calculated, while the others were measured values.

**Table 2:** Effects of YPF-P treatment on antioxidant capacity: MDA, malondialdehyde; SOD, superoxide dismutase; T-AOC, total antioxidant capacity, GSH-PX, glutathione peroxidase; ^a^, ^b^, ^c^ According to Tukey’s multiple comparisons, different superscripts in the same line indicate significant differences (*p* < 0.05).

Items	YPF-P Levels (g/kg Feed)				*p*-Value	
	0	1	2	4	Linear	Quadratic
T-AOC (U/mL)	0.90 ^b^	0.96 ^ab^	1.10 ^a^	0.98 ^ab^	0.072	0.056
SOD (U/L)	148.45 ^b^	234.78 ^a^	239.09 ^a^	241.02 ^a^	<0.001	<0.001
GSH-PX (U/mL)	1168.38 ^b^	1360.82 ^b^	1681.37 ^a^	1836.98 ^a^	<0.001	0.711
MDA (nmol/mL)	3.39 ^a^	2.43 ^b^	1.47 ^c^	1.12 ^c^	<0.001	0.137

## Data Availability

The data that support the findings of this study are available from the corresponding author upon reasonable request.
